# Combined assessment of clinical and patient factors on doctors’ decisions to prescribe antibiotics

**DOI:** 10.1186/s12875-016-0463-6

**Published:** 2016-06-03

**Authors:** Julia Strumiło, Sławomir Chlabicz, Barbara Pytel-Krolczuk, Ludmiła Marcinowicz, Dorota Rogowska-Szadkowska, Anna Justyna Milewska

**Affiliations:** Department of Family Medicine and Community Nursing, Medical University of Białystok, Mieszka I 4B, 15-054 Białystok, Poland; Department of Statistics and Medical Informatics, Medical University of Białystok, Białystok, Poland

**Keywords:** Antimicrobial agents, Physician–patient relations, Respiratory tract infections, Family physicians

## Abstract

**Background:**

Antibiotic overprescription is a worldwide problem. Decisions regarding antibiotic prescription for respiratory tract infections (RTIs) are influenced by medical and non-medical factors.

**Methods:**

In family medicine practices in Białystok, Poland, family medicine residents directly observed consultations with patients with RTI symptoms. The observing residents completed a questionnaire including patient data, clinical symptoms, diagnosis, any prescribed antibiotic, and assessment of ten patient pressure factors.

**Results:**

Of 1546 consultations of patients with RTIs, 54.26 % resulted in antibiotic prescription. Antibiotic prescription was strongly associated with rales (OR 26.90, 95 % CI 9.00–80.40), tonsillar exudates (OR 13.03, 95 % CI 7.10–23.80), and wheezing (OR 14.72, 95 % CI 7.70–28.10). The likelihood of antibiotic prescription was increased by a >7-day disease duration (OR 3.94, 95 % CI 2.80–5.50), purulent nasal discharge (OR 3.87, 95 % CI 2.40–6.10), starting self-medication with antibiotics (OR 4.11, 95 % CI 2.30–7.30), and direct request for antibiotics (OR 1.87, 95 % CI 1.30–2.80). Direct request not to prescribe antibiotics decreased the likelihood of receiving antibiotics (OR 0.34, 95 % CI 0.27–0.55).

**Conclusion:**

While clinical signs and symptoms principally impact prescribing decisions, patient factors also contribute. The most influential patient pressure factors were starting self-medication with antibiotics, and directly requesting antibiotic prescription or no antibiotic prescription. Interventions aiming to improve clinical sign and symptom interpretation and to help doctors resist direct patient pressure could be beneficial for reducing unnecessary antibiotic prescribing.

## Background

The majority of antibiotics prescriptions are issued in primary care for respiratory tract infections (RTIs) [1, 2]. Most RTIs do not require antibiotic therapy as they are predominantly of viral aetiology and are self-limited in nature [3, 4]. nevertheless, patients are often prescribed antibiotics unnecessarily and without clinical justification [5, 6]. Multiple interventions aiming at decreasing unnecessary antibiotics prescriptions for common RTIs have been targeted to doctors [7]. Such interventions have reportedly improved physicians’ antibiotic prescribing practices [8, 9], which consequently may slow resistance development and improve patient outcomes. A better understanding of the factors that influence general practitioners’ prescribing decisions could help in the design of more effective interventions to promote prudent antibiotic use.

While a doctor’s diagnostic skills are undoubtedly important in the prescribing process [10, 11], the motives for prescribing antibiotics seem to be more complex [12]. There are reportedly multiple reasons for unjustified prescription of antibiotics [13, 14], with pressure from patients and their families appearing to be an important factor [13, 14]. Patients use different types of direct and indirect pressure to influence physicians’ decisions concerning antibiotic prescription, which must be considered when planning interventions aiming to reduce unnecessary antibiotic prescriptions. Doctors may need to develop effective strategies to resist explicit or implicit requests to prescribe. On the other hand, some studies indicate that doctors often have false perceptions of their patients’ desires for antibiotics, and that patients do not want antibiotics as much as doctors think [15].

There has been much research regarding which factors influence antibiotics prescribing practices in ambulatory care [16]. However, most studies of doctor–patient interactions regarding antibiotic prescribing during RTI consultations are qualitative [17]. Systematic reviews report that the studies analysing antibiotics misprescription in ambulatory care are limited and heterogeneous, and that the available data are likely insufficient due to methodological limitations in a large number of these studies [16]. Moreover, quantitative studies are often based on simulated prescribing and do not permit examination of factors other than the doctor’s knowledge, as doctor–patient interactions influencing the prescription cannot be evaluated.

The present study aimed to examine real-life consultations with patients with RTIs in primary care in Poland. Questionnaires were used to record these consultations. Our main goal was to establish which symptoms and physical findings influenced the outcome of the consultation in terms of prescribing antibiotics, as well as the forms of patients’ direct or indirect requests for antibiotics.

## Methods

### Study design

This study was conducted in family doctors’ offices in Białystok—a city of approximately 300 000 inhabitants in north-eastern Poland—from 2007 to 2013. Białystok is so-called “green lungs of Poland”, a city with unpolluted, fresh air, compared to other Polish urban areas. It has a low level of occupational lung disease as Białystok is not an industrial city. Fifty family doctors with degrees in Family Medicine and fifty family medicine residents were voluntarily enrolled into the study and gave their consent to participate. The doctors were recruited through The Department of Family Medicine and Community Nursing from the Medical University of Białystok. Consultations with consecutive patients having any symptoms of respiratory infection were directly observed by the family medicine residents, who started their first year of 4-year family medicine training under the supervision of the fully qualified family medicine physicians. The family medicine trainees sat in the consultation, one-to-one with their supervisors. These trainees observed how the consultations of the patients with RTIs were managed by senior doctors and completed questionnaires during or immediately after the consultation, irrespective of whether an antibiotic was prescribed.

### Measurements

On the questionnaire, the resident recorded the patient’s age, gender, and symptoms. The first part of the questionnaire also included questions regarding common physical findings (e.g. enlarged lymphatic nodes or auscultation sounds), common additional tests, probable diagnosis, and whether and what kind of antibiotic was prescribed for the patient. Each patient was given only one diagnosis, classified according to International Classification of Diseases (ICD-10). Modifications included that acute pharyngitis was divided into two categories (viral and bacterial), and that the additional diagnosis of unspecified bacterial superinfection was provided.

The second part of the questionnaire covered ten patient factors relating to antibiotic prescription (presented in a table below) and the resident’s general impression of whether the patient pressured the physician for antibiotic prescription, if no pressure was present, or it was unclear to the observer whether any pressure was present. For each patient factor, the questionnaire included open space for the observing resident to write the exact words that the patient used to pressure the doctor for prescription, if the resident deemed it necessary or of interest.

**Table:** 

Patient factor	Example
Patient factor 1: patient started treatment on his own, as he/she was in possession of an antibiotic (asked in the pharmacy, had at home, other source, what source?)	“I had antibiotics from the previous therapy and I started them on my own.”
Patient factor 2: direct request for antibiotics	“I need an antibiotic as I have had several infections in the last few weeks.”
Patient factor 3: candidate diagnosis	“I must have sinusitis.” “This must be tonsillitis.”
Patient factor 4: emphasising the necessity of quick recovery, appealing to life circumstances	“I need to get back to work.” “I need to take care of my child.”
Patient factor 5: without being asked by the physician, the patient insists that antibiotics were effective in similar cases in the past	“When I had similar symptoms last year, I received an antibiotic and it helped.”
Patient factor 6: the patient recalls that a family member was prescribed an antibiotic when he/she had similar symptoms and had good results	“My mom was prescribed an antibiotic when she had a similar cough and it helped.”
Patient factor 7: the patient says that similar symptoms in the past did not resolve without an antibiotic	“I know that when I have cough like this it won’t stop unless I get an antibiotic. It is always like that.”
Patient factor 8: patient emphasizes the severity of symptoms (portraying severity of illness)	“This cough is going to kill me.” “The sore throat is so intense.”
Patient factor 9: direct request not to prescribe antibiotics	“I don’t want an antibiotic as it makes my immune system weaker.”
Patient factor 10: other expressions used by the patient that may influence prescription decision	“Over-the-counter remedies that I took didn’t help at all and neither did the syrup you prescribed me.”

### Selection of study subjects

The patients whose data was collected anonymously were consecutive patients coming for consultation for RTI symptoms, the participating patients were identified when they entered the doctors’ office and declared that they are willing to have a consultation with their family physician because of the RTI symptoms. The patients attending primary care agreed to have their data collected by the trainee present during their consultation. All data were stored anonymously. The study protocol was approved by the Bioethical Committee of the Medical University of Białystok.

### Outcomes and statistical analysis

Univariate logistic regression was performed for „antibiotic prescription” with variables such as patient’s symptoms and physical findings and patient pressure factors as independent variables. Solely the factors that were found statistically significant in the univariate regression model were included into the multivariate regression model. Next, the method of backward elimination was applied, the essence of which was to eliminate the predictor with the smallest impact in the next steps until the satisfactory quality of parameters of the model was obtained.

We presented results for all analysed independent variables (both statistically significant and insignificant) when analysis was performed by the method of univariate regression. We also presented all variables, that were found in the created multivariate statistical regression model (all of them were statistically significant). P value of <0.05 was considered statistically significant. STATA/1C 12.1 was used for analyses (StataCorp LP, Texas, USA).

## Results

A total of 1456 visits of patients with RTIs were registered, of whom 774 were female (53.16 %) and 682 were male (46.84 %). Table 1 presents the age distribution of the participating patients. The median age was 29 years, with a range of 1 month to 91 years. Children under the age of 18 visited the physician with their parents who acted on child’s behalf. The population structure was typical of the Polish primary care setting in which a physician provides care for both children and adults.

**Table 1:** Patient age distribution (*N =* 1456)

Age in years	Number of patients (n)	Percentage (%)
0–10	292	20.05
10–20	189	12.98
20–30	251	17.23
30–40	249	17.10
40–50	187	12.84
50–60	154	10.58
60–70	82	5.63
70–80	40	2.74
80–90	11	0.75
90–100	1	0.07

For the majority of patients (1250 patients; 85.85 %), the recorded consultation was their first visit to the family doctor’s office with symptoms of their present respiratory tract infection. However, for 206 patients (14.15 %), the recorded consultation was their second visit regarding these symptoms for the same RTI. Of the patients, 1035 (71.00 %) reported a duration of symptoms that was shorter than seven days, while 421 patients (28.91 %) reported that their disease had lasted longer than seven days. The most commonly reported RTI symptoms were sore throat (63.12 %), cough (71.22 %), and rhinorrhoea (54.05 %). The most common diagnoses were viral infection (538 patients; 36.95 % of visits), acute pharyngitis/tonsillitis of probable bacterial aetiology (270 patients; 18.54 % of visits), acute bronchitis (143 patients; 9.82 % of visits), and acute pharyngitis/tonsillitis of probable viral aetiology (138 patients; 9.48 % of visits). The least common diagnosis was chronic bronchitis exacerbation (26 patients; 1.79 % of visits).

Antibiotics were prescribed in the consultations of 790 patients (54.26 % of all consultations), while 44.85 % of RTI consultations ended without antibiotic prescription. Antibiotics were more frequently prescribed if the patient had consulted the doctor more than once for the same RTI. A total of 635 patients (50.84 %) were given an antibiotic prescription during their first visit for an RTI, whereas 80.98 % of patients (*n* = 166) received an antibiotic prescription when seeing a doctor for the second time for the same RTI. Eleven patients (0.76 %) were prescribed locally acting antibiotics—including fusafungine, which was available in pharmacies at the beginning of the study (2007), or gentamicin via nebulisation, although this is not recommended in the treatment guidelines. Two patients (0.14 %) were referred to the hospital. Almost all decisions were made on empirical grounds. In 76 consultations (5.22 %) additional testing was performed, including culture in 14 consultations (0.96 % of visits), chest X-ray in 32 consultations (2.2 % of visits), lab tests (ESR, CRP, and WBC) in 24 consultations (1.65 % of visits), and serology in 6 consultations (0.41 % of visits).

The observers noted no patient pressure factors in 712 consultations (48.9 %), one patient pressure factor in 336 consultations (23.7 %), two patient pressure factors in 166 consultations (11.4 %), three patient pressure factors in 127 consultations (8.7 %), and eight patient pressure factors in 2 consultations (0.14 %). The most common patient pressure factor was candidate diagnosis—for example, “I must have bronchitis”—which was reported in 308 consultations (21.15 %). The second most frequent patient pressure factor was emphasizing the severity of illness and the patient’s discomfort, which was observed in 272 consultations (18.68 %). In these cases, the patients made comments such as “This cough is going to kill me” or “The sore throat is so intense” implying that the patient was in prompt need of the doctor’s help. The third most common patient pressure factor was a direct request to prescribe antibiotics, which occurred in 247 of consultations (16.96 %). On the other hand, as many as 80 patients (5.49 %) directly asked the physician not to prescribe antibiotics.

Univariate logistic regression (*N =* 1454) revealed that some factors influenced antibiotic prescribing behaviour, both significantly and insignificantly, with statistical significance indicated by *P* < 0.05 (Table 2). Table 3 shows only the factors that were significantly linked to antibiotic prescribing (*P* < 0.05) based on analysis by multivariate logistic regression.

**Table 2:** Results of univariate logistic regression model for ‘’antibiotic prescription”. Patient symptoms and physical findings, and patient pressure factors and their influence on prescribing decision according to univariate logistic regression (*N =* 1454)

Variables	Number of patients (%) *N =* 1454	Odds Ratio	*P*	95 % Confidence Interval
Temperature above 38 °C for at least one day	659 (45.26 %)	2.12	<0.001	1.72–2.62
Symptom duration longer than 7 days	421 (28.91 %)	3.68	<0.001	2.85–4.75
Headache while bending forward	181 (12.43 %)	1.76	0.001	1.27–2.44
Redness of the throat	556 (38.19 %)	1.72	<0.001	1.38–2.13
Tonsillar exudates	178 (12.23 %)	13.96	<0.001	7.69–25.34
Enlargement of cervical and/or submandibular lymph nodes	260 (17.86 %)	3.13	<0.001	2.30–4.25
Expectoration of purulent sputum	203 (13.94 %)	4.83	<0.001	3.20–7.10
Thick purulent nasal discharge	173 (11.88 %)	3.02	<0.001	2.09–4.38
Auscultation sounds: wheezing and rhonchi	181 (12.43 %)	11.03	<0.001	6.43–18.93
Auscultation sounds: rales or crepitations	99 (6.80 %)	21.57	<0.001	<0.001 7.89–59.00
Patient factor 1: patient started treatment on his own as he/she was in possession of an antibiotic	111 (7.62 %)	3.19	<0.001	2.00–5.08
Patient factor 2: direct request for antibiotics	247 (16.96 %)	2.16	<0.001	1.60–2.89
Patient factor 3: candidate diagnosis	308 (21.15 %)	1.41	0.009	1.08–1.82
Patient factor 5: the patient insists that antibiotics were effective in previous similar cases	115 (7.90 %)	1.29	0.195	0.88–1.91
Patient factor 6: the patient recalls that a family member was prescribed an antibiotic when he/she had similar symptoms and had good results	92 (6.32 %)	1.17	0.473	0.76–1.80
Patient factor 7: the patient says that similar symptoms in the past did not resolve without an antibiotic	103 (7.07 %)	1.30	0.200	0.87–1.97
Patient factor 8: patient’s emphasis on symptom severity (portraying severity of illness)	272 (18.68 %)	1.88	<0.001	1.40–2.48
Patient factor 10: other expressions used by the patient that may influence prescription decision	105 (7.21 %)	1.77	0.008	1.16–2.70
Factors that decreased the likelihood of antibiotic prescription				
Sore throat	919 (63.12 %)	0.65	<0.001	0.52–0.80
Hoarseness	337 (23.15 %)	0.74	0.014	0.58–0.94
Cough	1037 (71.22 %)	0.59	<0.001	0.47–0.75
Rhinorrhoea	787 (54.05 %)	O.26	<0.001	0.20–0.32
Patient factor 9: direct request not to prescribe antibiotics	80 (5.49 %)	0.35	<0.001	0.22–0.57
Patient factor 4: emphasising the necessity of quick recovery/appealing to life circumstances	184 (12.64 %)	0.97	0.829	0.71–1.32

**Table 3:** Results of multivariate logistic regression model for “antibiotic prescription”. Factors significantly associated with antibiotic prescription at consultation according to multivariate logistic regression (*N =* 1454)

Variables	Number of patients *N* Percentage (%) *N =* 1454	Odds Ratio	*P*	95 % Confidence Interval
Temperature above 38 °C (for at least one day)	659 (45.26 %)	1.42	0.019	1.06–1.90
Symptom duration longer than 7 days	421 (28.91 %)	3.94	<0.001	2.83–5.48
Headache while bending forward	181 (12.43 %)	1.93	0.003	1.26–2.95
Redness of the throat	556 (38.19 %)	2.26	<0.001	1.63–3.12
Tonsillar exudates	178 (12.23 %)	14.72	<0.001	7.68–28.18
Enlargement of cervical and/or submandibular lymph nodes	260 (17.86 %)	2.74	<0.001	1.81–4.13
Expectoration of purulent sputum	203 (13.94 %)	2.22	0.001	1.36–3.63
Thick purulent nasal discharge	173 (11.88 %)	3.87	<0.001	2.43–6.15
Auscultation sounds: wheezing or rhonchi	181 (12.43 %)	13.03	<0.001	7.14–23.8
Auscultation sounds: rales or crepitations	99 (6.80 %)	26.90	<0.001	9.00–80.35
Patient factor 1: starting antibiotics on his/her own	111 (7.62 %)	4.11	<0.001	2.33–7.27
Patient factor 2: direct request for antibiotics	247 (16.96 %)	1.87	0.002	1.27–2.77
Factors that decreased the likelihood of antibiotic prescription				
Patient factor 9: direct request not to prescribe	80 (5.49 %)	0.34	0.001	0.18–0.64
Sore throat	919 (63.12 %)	0.58	0.001	0.42–0.80
Rhinorrhoea	787 (54.05 %)	0.36	<0.001	0.27–0.48

Antibiotic prescription was most strongly associated with abnormalities in physical examination—such as rales, which showed an adjusted odds ratio (OR) of 26.90 and a 95 % confidence interval (CI) of 9.00–80.40; tonsillar exudates (OR 14.72, CI 7.70–28.10); and wheezing (OR 13.03, CI 7.10–23.80). A disease duration of longer than seven days increased the chance of antibiotic prescription almost 4-fold (OR 3.94, CI 2.80–5.50). Antibiotic prescription was associated with the presence of purulent nasal discharge (OR 3.87, CI 2.40–6.10), while the presence of rhinorrhoea decreased the likelihood of receiving antibiotics by about 3-fold (OR 0.36, CI 0.27–0.48). Patient pressure factors 1 (the patient started antibiotic on his own as he/she was already in possession) and 2 (direct request for antibiotics) were both associated independently with antibiotic prescription: adjusted odds ratios of 4.11 (CI 2.30–7.30) and 1.87 (CI 1.30–2.80), respectively. On the other hand, patient pressure factor 9 (direct request not to prescribe antibiotics) decreased the chance of receiving antibiotics by about 3-fold (adjusted OR 0.34, CI 0.27–0.55) (Table 3).

## Discussion

The present results confirmed a high rate of antibiotic prescription (54.26 %), even though the majority of diagnoses made by family physicians suggested a viral aetiology. A 2002 study of family physicians’ prescription choices in the same region of Poland showed an even higher percentage of patients receiving antibiotic prescriptions, with more than 60.00 % of consultations ending with antibiotic prescription, including 90.00 % of patients diagnosed with acute bronchitis [18]. Studies from other countries have also reported that patients are prescribed antibiotics even when their diagnoses indicate viral causes [13, 19]. For example, in a paediatric study from Italy, 22.00 % of children with RTIs due to viral causes received antibiotic prescriptions [20]. This supports that factors other than clinical findings contribute to prescription decisions in cases of RTI, with patient expectation seeming to be a particularly strong factor [20].

Moreover, even physical findings are sometimes clearly misinterpreted. It is not surprising that rales and tonsillar exudates greatly increased the chance of antibiotic prescription, as these findings may indicate bacterial disease. However, wheezing or rhonchi also significantly increased the probability of antibiotic prescription (OR 13.03, CI 7.14–23.80), even though about 95 % of cases of acute bronchitis are attributed to viruses. Routine antibiotic treatment for acute bronchitis is not recommended [3], but overuse of antibiotics is very common in such cases and is a serious worldwide problem.

Our results also confirmed an association between symptom duration of longer than seven days and antibiotic prescription. Some studies have reported this to be the most important factor influencing antibiotics prescribing rates [21], even though 1 to 2 weeks is a normal duration for an uncomplicated respiratory tract infection and complications develop in only a very small percentage of cases [22]. Here we also found that purulent manifestations, including coloured nasal discharge and expectoration of purulent sputum, were associated with higher prescription rates, as has been noted in other studies [23–25]. However, the presence of purulent manifestations is not a reliable way to distinguish between infections of viral and bacterial origin [24] as coloured discharge is commonly found in uncomplicated RTIs and there is no significant difference in outcomes between patients with or without purulent discharge [22]. Finally, red throat and headache while bending forward increased the likelihood of antibiotic prescription by about two-fold each, even though these symptoms do not indicate bacterial infection.

These observed liberal prescribing practices might stem from the general practitioners’ lack of knowledge or uncertainty regarding diagnosis, as accessory investigations are rarely performed in family medicine in Poland. In our study, additional testing was completed in about 5.22 % of cases and most decisions were of an empirical nature, even though current Polish guidelines recommend to diagnose bacterial pharyngitis/tonsillitis based on streptococcal test or culture, and pneumonia in adults—based on abnormalities found in the chest X-ray [26]. Other studies have also reported infrequent use of additional testing in RTIs in Poland [27], which may result from the financing of primary care in Poland where doctors bear the costs of accessory testing. Diagnostic uncertainty based on clinical observations has been recognized as a factor influencing unjustified antibiotic prescribing [28]. If accessory investigations (e.g. CRP testing) were more regularly performed in Poland, it is likely that many unnecessary antibiotic therapies could be avoided—as numerous studies show that CRP testing leads to reduced antibiotic prescribing [29]. Without additional testing in individual cases it is quite challenging to say with any degree of certainty whether pharyngitis or tonsillitis and other RTIs are of bacterial, viral or mixed aetiology. However, it seems that family physicians might benefit from further education regarding interpretation of clinical signs and symptoms, for example that wheezing, purulent nasal discharge and the duration of illness longer than 7 days should not necessarily lead to antibiotic prescription as those symptoms and clinical findings do not imply bacterial disease.

Another important factor is patient pressure [13]. Patients often have insufficient knowledge regarding aetiology of RTIs and antibiotics. For example, it was reported that 41 % of Italian parents from Emilia-Romagna assume that bacteria is a cause of the common cold [20]. Our present findings confirmed that patient expectations for antibiotic prescription played an important role in the doctors’ prescribing decisions. It seems that in Poland it is quite common for patients to possess and to start self-medicating with antibiotics (7.62 % of patients in our study), and this situation increased the patient’s likelihood of obtaining a prescription from a doctor by about four-fold. Such patients often had antibiotics remaining from previous therapies, had obtained them from family and friends, or had even received them from the pharmacy without a prescription—which is illegal since, unlike in Belarus or Georgia, antibiotics are not available over the counter in Poland. These results might indicate easy access to antibiotics in Poland, as well as the patients’ readiness to self-medicate. The continuation of antibiotic therapy by the doctor may reflect eagerness to meet patient expectations and to avoid potential conflict in order to not lose patients to other doctors. In Poland, doctors are not monitored for the quantity and quality of antibiotic prescriptions.

Regarding other forms of patient pressure, direct request for antibiotics led to a greater likelihood of receiving antibiotics. However, other patient pressure strategies—including candidate diagnosis, portraying the severity of illness, and appealing to life circumstances—were not significantly associated with antibiotic prescription. For example candidate diagnosis: “I must have bronchitis” does not necessarily lead to antibiotic prescription, as the doctor—unlike the patient—is aware that bronchitis is most often a viral disease and can explain the aetiology and self-limiting nature of bronchitis to his patient. Direct request not to prescribe antibiotics also influenced prescription decision greatly, as it decreased the chance of prescription by about three-fold. Direct requests to prescribe or not to prescribe likely influenced consultations the most because family physicians tried to avoid conflict. Moreover, the physicians may not have been comfortable/self-assured enough to resist direct patient pressure and to manage the consultation assertively. It may also be difficult to reassure the patient that no antibiotic is needed without accessory tests.

These findings could indicate the need to implement interventions helping doctors to communicate their decisions to patients when they are opposite to the patients’ expectations, as well as to reduce doctors’ fear of potential conflict with patients when not meeting their perceived needs [17]. One of such interventions based on GPs receiving web-based training carried out in six European countries (including Poland) was shown to be effective in terms of increasing GPs confidence and positive change in family doctors’ and patients’ attitudes towards prescribing antibiotics [30]. Some other articles also confirm that communication training for health care professionals can be effective in order to reduce antibiotic prescribing in RTIs [31, 32].

It should also be noted that numerous studies suggest that doctors’ perceptions of patients’ needs may not be accurate. When patients ask for antibiotics, they sometimes really want an accurate explanation, concern about their symptoms, and reassurance [33], and will be satisfied with these responses regardless of whether a prescription is written [34]. Some studies reveal insufficient public knowledge about antibiotics, as patients believe that antibiotics are of benefit in viral infections [20, 35]. Therefore, careful explanation of the nature of the disease and of the treatment provided by the healthcare professional may be sufficient, and may even contribute more to patient satisfaction than prescribing antibiotics as a form of coping strategy.

Our study has several limitations. Firstly, all of the included doctors were qualified family physicians—the sample of participating doctors was not random—and their prescribing pattern could be different from those of other doctors working in primary care in Poland that can be a source of sample selection bias bias, especially the fact that our study doctors are probably more up-to-date with the guidelines and their prescribing pattern is more appropriate. Secondly, the doctors’ prescribing behaviour could have been influenced by being observed by family medicine residents and social desirability bias could occur which could result in prescribing less antibiotics than usual. Thirdly, the questionnaires were filled in by the observing residents, who recorded their supervisor’s practice and were not validated against the patients’ records. The apprenticeship training model and fundamental power disproportion may have led to potential information bias with the result of omitting inappropriate behaviours of the observed doctors. Moreover, the information recorded regarding the types of patient pressure was subjective, as the observing resident assessed the patient pressure categories according to their personal impressions from the consultation which could potentially lead to observer-expectancy bias and obtain different results when different observers observed the same event but their personal characteristics and expectations influenced the recording. An example of that could be classifying the same patient request for antibiotics into different patient pressure categories by two different observers. This was not measured in any objective way.

## Conclusions

Clinical signs and symptoms play a major role in prescribing decisions. However, patient pressure factors also contribute, with the most effective types of pressure being when a patient has already started self-medication with antibiotics as well as direct requests to prescribe or not to prescribe. Our present findings will provide a better understanding of the factors influencing the antibiotic prescription decision-making process, and could be used to guide interventions aiming to improve doctors’ assertiveness and communication skills in order to decrease unnecessary prescribing.

## Abbreviations

CI, confidence interval; GPs, general practitioners; OR, odds ratio; RTI, respiratory tract infection
